# The *Lysobacter capsici* AZ78 Genome Has a Gene Pool Enabling it to Interact Successfully with Phytopathogenic Microorganisms and Environmental Factors

**DOI:** 10.3389/fmicb.2016.00096

**Published:** 2016-02-05

**Authors:** Gerardo Puopolo, Selena Tomada, Paolo Sonego, Marco Moretto, Kristof Engelen, Michele Perazzolli, Ilaria Pertot

**Affiliations:** ^1^Department of Sustainable Agro-Ecosystems and Bioresources, Research and Innovation Centre, Fondazione Edmund MachSan Michele all'Adige, Italy; ^2^Department of Agricultural and Environmental Science (DISA), PhD School of Agricultural Science and Biotechnology, University of UdineUdine, Italy; ^3^Department of Computational Biology, Research and Innovation Centre, Fondazione Edmund MachSan Michele all'Adige, Italy

**Keywords:** Lysobacter, biological control, lytic enzymes, siderophores, environmental stress

## Abstract

*Lysobacter capsici* AZ78 has considerable potential for biocontrol of phytopathogenic microorganisms. However, lack of information about genetic cues regarding its biological characteristics may slow down its exploitation as a biofungicide. In order to obtain a comprehensive overview of genetic features, the *L. capsici* AZ78 genome was sequenced, annotated and compared with the phylogenetically related pathogens *Stenotrophomonas malthophilia* K729a and *Xanthomonas campestris* pv. *campestris* ATCC 33913. Whole genome comparison, supported by functional analysis, indicated that *L. capsici* AZ78 has a larger number of genes responsible for interaction with phytopathogens and environmental stress than *S. malthophilia* K729a and *X. c*. pv. *campestris* ATCC 33913. Genes involved in the production of antibiotics, lytic enzymes and siderophores were specific for *L. capsici* AZ78, as well as genes involved in resistance to antibiotics, environmental stressors, fungicides and heavy metals. The *L. capsici* AZ78 genome did not encompass genes involved in infection of humans and plants included in the *S. malthophilia* K729a and *X. c*. pv. *campestris* ATCC 33913 genomes, respectively. The *L. capsici* AZ78 genome provides a genetic framework for detailed analysis of other *L. capsici* members and the development of novel biofungicides based on this bacterial strain.

## Introduction

Genome sequencing represents an excellent tool for biological characterization of bacterial species; especially in the case of species that have been largely underexplored. In the family Xanthomonadaceae (Saddler and Bradbury, 2005), attention has been paid particularly to members that are pathogenic to humans (*Stenotrophomonas*) and plants (*Xanthomonas* and *Xylella*; Simpson et al., 2000; da Silva et al., 2002; Crossman et al., 2008). In contrast, other bacterial genera have been underexplored, as in the case of the genus *Lysobacter*, which was established in 1978 (Christensen and Cook, 1978). Since most *Lysobacter* spp. were wrongly classified as *Myxobacteriales* and *Cythophagales*, and several *Lysobacter* strains were wrongly assigned to *Stenotrophomonas* and *Xanthomonas* spp. (Christensen and Cook, 1978; Giesler and Yuen, 1998; Sakka et al., 1998; Nakayama et al., 1999), the importance of this genus was underestimated for a long time. The increasing number of 16S rDNA gene sequences available in public databases and the polyphasic approach to the identification of bacterial strains has led to an increase in the identification of new *Lysobacter* species. So far the genus has expanded to include 37 species (Singh et al., 2015) from the initial four: *L. antibioticus, L. brunescens, Lysobacter enzymogenes*, and *L. gummosus* (Christensen and Cook, 1978).

Some bacterial strains of the *Lysobacter* species act as biological control agents (BCAs) of plant diseases (Kobayashi and Yuen, 2007; Hayward et al., 2010). To date, most of the BCAs characterized have belonged to *L. enzymogenes* (Folman et al., 2003; Sullivan et al., 2003; Qian et al., 2009). Antagonistic mechanisms have received most attention in recent years, with the production of antibiotics and lytic enzymes by *L. enzymogenes* 3.1T8, C3, and OH11 and the related regulatory mechanisms being studied in some of these bacterial strains (Folman et al., 2004; Kobayashi et al., 2005; Palumbo et al., 2005; Yu et al., 2007; Zhang et al., 2011; Qian et al., 2012, 2013). Similarly to *L. enzymogenes, L. capsici* strains possess characteristics exploitable for the control of phytopathogenic microorganisms (Park et al., 2008). For example, the type strain *L. capsici* YC5194 produces secondary metabolites that inhibit the growth of phytopathogenic fungi (Park et al., 2008) and the *L. capsici* strain PG4 controls tomato foot and root rot caused by *Fusarium oxysporum* f. sp. *radicis-lycopersici* (Puopolo et al., 2010). Some *L. capsici* strains have been isolated from soils suppressive to *Rhizoctonia solani* and have been shown to be involved in the control of other phytopathogenic fungi (Postma et al., 2010). Other *L. capsici* strains can control nematodes, as in the case of *L. capsici* YS1275, used against *Meloidogyne incognita* (Lee et al., 2014), or oomycetes, as in the case of *L. capsici* AZ78 (*Lc* AZ78) used to control *Phytophthora (P.) infestans* and *Plasmopara (Pl.) viticola* (Puopolo et al., 2014a,b), indicating their high potential as broad spectrum BCAs. *Lc* AZ78's resistance to copper is an additional positive feature for a BCA, because it can be integrated within plant protection strategies including the use of copper fungicides (Puopolo and Pertot, 2014).

In comparison to *L. enzymogenes*, much less is known about the biological features of *L. capsici* (Puopolo et al., 2015). As understanding the biological characteristics of a microorganism is crucial for its development as a biopesticide, we sequenced the *Lc* AZ78 genome using PacBio technology and carried out functional experiments to assess the biological properties predicted by the genome analysis. To obtain a comprehensive overview of the genetic cues of *L. capsici*-specific biological characteristics, we compared the *Lc* AZ78 genome with the genome of two phylogenetically similar bacteria (Kobayashi and Yuen, 2007; Hayward et al., 2010): the opportunistic human pathogen *S. malthophilia* K729a (*Sm* K729a) and the phytopathogen *X. campestris* pv. *campestris* ATCC 33913 (*Xcc* ATCC 33913).

## Materials and methods

### Microorganisms

*L. capsici* AZ78 was stored at length in glycerol 40% at −80°C and routinely grown on Luria-Bertani Agar at 27°C (LBA, Sigma-Aldrich, USA). In all the experiments *Lc* AZ78 cell suspensions were prepared by flooding LBA dishes with 5 ml of sterile saline solution (0.85% NaCl) after 72 h growth at 27°C. *L. capsici* AZ78 cells were then scraped from the medium surface using sterile spatulas and collected in sterile 15 ml tubes. The resulting *Lc* AZ78 cell suspensions were centrifuged (11,200 *g*, 5 min) and the pelleted cells were suspended in sterile distilled water to a final absorbance of 0.1 at 600 nm, corresponding to 1 × 10^8^ Colony Forming Units (CFU)/ml. *L. capsici* AZ78 was used at this concentration in all experiments, except when otherwise indicated.

The phytopathogenic bacteria and fungi used in this work (Table S1) were grown respectively on Nutrient Agar (NA, Oxoid, United Kingdom) at 28°C and Potato Dextrose Agar (PDA, Oxoid) at 25°C. Bacterial strains were stored at length in glycerol 40% at −80°C, while fungal strains were stored on PDA slants at room temperature. *P. infestans* isolate was maintained on Pea Agar Medium (PAM, 12.5% frozen peas and 1.2% agar in distilled water) at 17°C and stored at length in glycerol 20% at −80°C.

### DNA extraction, genome sequencing, and assembly

*L. capsici* AZ78 genomic DNA was extracted with a PureLink Genomic DNA Mini Kit (Thermo Fisher Scientific, Invitrogen, USA) according to the manufacturer's instructions. Once extracted, DNA integrity and the absence of RNA contamination was checked on a 1% agarose gel. Subsequently, the whole genomic DNA of *Lc* AZ78 was sequenced using PacBio technology at Baseclear B.V. (Leiden, Netherlands). A 10-kb PacBio single-molecule real-time (SMRT) cell was employed (Chin et al., 2013). The generated subreads were *de novo* assembled using the RS hierarchical genome assembly process (HGAP) protocol version 3.0, as available in SMRT Portal v2.0 (https://github.com/PacificBiosciences/Bioinformatics-Training/wiki/HGAP-in-SMRT-Analysis). The SMRT Portal was configured and used with a public machine image that Pacific Biosciences maintains and upgrades on Amazon Cloud (https://github.com/PacificBiosciences/Bioinformatics-Training/wiki/%22Installing%22-SMRT-Portal-the-easy-way—Launching-A-SMRT-Portal-AMI).

This Whole Genome Shotgun project has been deposited at DDBJ/EMBL/GenBank under the accession no. JAJA00000000. The version described in this paper is version JAJA02000000.

### Genome annotation and comparative analysis

The genome of *Lc* AZ78 was annotated with the online platform Rapid Annotation using Subsystem Technology (RAST) version 2.0 (Aziz et al., 2008). For genome comparison, the genomes of *Sm* K729a (AM743169) and *Xcc* ATCC 33913 (AE008922) were submitted to the same online platform to eliminate bias deriving from the different annotation systems employed. Genetic content comparison between genomes was conducted at nucleotide and amino acid level using BLASTN and BLASTP, respectively. Both minimum length > 70 and identity ≥ 70% at amino acid level were used as threshold parameters.

### Proteolytic activity in the interaction between *Lysobacter Capsici* AZ78 and *Phytophthora infestans*

Plugs (5 mm) were cut from the edge of 7-day-old *P. infestans* colonies and transferred onto cellophane film overlying PAM dishes and incubated at 20°C for 7 days. After the incubation period, *P. infestans* macrocolonies originating from the plugs were transferred into 2 ml sterile tubes containing 500 μl of Phosphate Buffer Solution (PBS, 0.8% NaCl; 0.02% KCl; 0.145% NaH_2_PO_4_; 0.025% KH_2_PO_4_). Subsequently, the tubes were inoculated with 50 μl of a *Lc* AZ78 cell suspension (1 × 10^9^ CFU/ml). Tubes containing PBS, PBS with *Lc* AZ78 and PBS with *P. infestans* were used as controls.

All the tubes were incubated at 25°C for 48 h and were processed at 6, 24, and 48 h to determine proteolytic activity. Briefly, tubes were centrifuged (16,100 *g*, 5 min) and 225 μl of supernatants were mixed with 150 μl of 1% Casein stock solution (50 mM Tris-HCL, pH 8.8) in new sterile 2 ml tubes. Subsequently, the tubes were incubated at 37°C for 1 h and undigested substrates were precipitated by adding 375 μl of 5% Trichloroacetic acid. The tubes were then centrifuged at 16,100 *g* for 3 min. The resulting supernatants were transferred into new sterile 2 ml tubes containing 400 μl of 1 M NaOH, and absorbance at 405 nm (A_OD405 nm_) was assessed using a spectrophotometer. At each time point, three 2 ml tubes (replicates) for each treatment were used, and the experiment was repeated.

### Production of lytic enzymes and siderophores

*L. capsici* AZ78 was evaluated in terms of its ability to degrade cellulose, chitin, laminarin and proteins using classic methods (Cowan, 1974; Sambrook and Russell, 2001). The occurrence of a clear halo surrounding *Lc* AZ78 colonies was checked after 48 h incubation at 27°C.

To determine siderophore production, LBA dishes were overlaid with CAS agar medium (Schwyn and Neilands, 1987). The final medium looked dark blue. Five microliter of *Lc* AZ78 cell suspension were spot inoculated onto these dishes. Siderophore production associated with the change in the color of CAS agar medium (Schwyn and Neilands, 1987) was assessed after 72 h incubation at 27°C.

### *In vitro* antifungal and antibacterial activity

The antifungal activity of *Lc* AZ78 against 23 phytopathogenic fungi (Table S1) was evaluated by using the classic dual-culture method. Briefly, 50 μl of *Lc* AZ78 cell suspension were spotted on two opposite edges of a PDA plate. After 24 h incubation at 27°C, plugs of mycelium (5 mm) were cut from the edge of young fungal colonies grown on PDA and placed at the center of the plates containing the *Lc* AZ78 macrocolonies. PDA plates seeded only with mycelium plugs were used as controls. After 4 days incubation at 25°C, inhibition of mycelial growth was evaluated by scoring the diameters of fungal colonies. Each test was performed in triplicate and the experiment was repeated.

*In vitro* antibacterial activity of *Lc* AZ78 against eight phytopathogenic bacteria (Table S1) was evaluated. NA dishes were spot inoculated with 50 μl of *Lc* AZ78 cell suspension and incubated for 72 h at 27°C. *L. capsici* AZ78 cells were then killed by exposure to chloroform vapor for 60 min. The plates were subsequently aerated under the laminar flow for 60 min. Dishes were overlaid with 8 ml of 0.4% agar PBS, mixed with 2 ml of a suspension containing 1 × 10^8^ CFU/ml of the test bacterial strains. NA dishes not seeded with *Lc* AZ78 and NA dishes overlaid with 0.4% agar PBS only were used as controls. Each test was performed in triplicate and the experiment was repeated. The diameters of inhibition haloes were scored after 48 h incubation at 28°C.

### Determination of *Lysobacter capsici* AZ78 resistance to Cobalt and Zinc

To determine the ability of *Lc* AZ78 to resist heavy metals, *Lc* AZ78 cells were grown on LBA amended with CoCl_2_ and ZnSO_4_ (Sigma-Aldrich). Briefly, filter-sterilized CoCl_2_ and ZnSO_4_ solutions were added to LBA to obtain the final concentrations of 0.5 and 1 mM, respectively. Subsequently, 100 μl of a serial dilution, from 10^−1^ to 10^−7^, of *Lc* AZ78 cell suspension were spread onto LBA and LBA amended with CoCl_2_ and ZnSO_4_ using sterile spatulas. *L. capsici* AZ78 CFU were counted after 4 days of incubation at 27°C. Three replicates (Petri dishes) of each combination (dilution and heavy metal concentration) were prepared and the experiments were repeated. *Bacillus amyloliquefaciens* FZB42 was used as control. The number of *Lc* AZ78 and *B. amyloliquefaciens* FZB42 CFU were log_10_ transformed before statistical analysis.

### Resistance of *Lysobacter capsici* AZ78 to chemical fungicides and insecticides

Thirty-two plant protection products commonly applied for the chemical control of grapevine plant diseases were used in this experiment (Table S2). Each plant protection product was dissolved in distilled water, filter-sterilized and added to LBA to achieve the maximum concentration commonly applied in the field (Table S2). A volume of 100 μl of *Lc* AZ78 cell suspension (1 × 10^3^ CFU/ml) was spread onto LBA and LBA amended with plant protection products with sterile spatulas. *L. capsici* AZ78 CFUs were counted after 72 h of incubation at 27°C. Viability reduction was calculated according to this formula:

(CFU grown on LBA – CFU grown on LBA amended with the plant protection product) / (CFU grown on LBA)

Three Petri dishes (replicates) were used for each plant protection product, and the experiments were repeated.

### Sensitivity of *Lysobacter capsici* AZ78 to antibiotics

The standardized disc susceptibility testing method (Andrews et al., 2011) was used to determine the sensitivity of *Lc* AZ78 to ampicillin, chloramphenicol, erythromycin, gentamicin, kanamycin, streptomycin, tetracycline, tobramycin, trimethoprim, and vancomycin. Briefly, Mueller Hinton Agar (MHA, Sigma-Aldrich) was poured into sterile Petri dishes to reach a depth of 4 mm (Atlas, 2004). A volume of 100 μl of *Lc* AZ78 cell suspension (2 × 10^8^ CFU/ml) was spread over the entire surface of MHA dishes using sterile spatulas. Subsequently, three discs of the same antibiotic (Oxoid) were placed on each inoculated Petri dish. After 48 h incubation at 27°C, the diameters of the inhibition zones were measured with a caliper. Three Petri dishes (replicates) were used for each antibiotic and the experiments were repeated. Resistance or sensitivity to antibiotics was assigned on the basis of the inhibition zone diameters (Andrews et al., 2011; Anonymous, 2011).

### Statistical analysis

The data obtained in the experiments aimed at assessing (i) proteolytic activity; (ii) antifungal and antibacterial activity; (iii) resistance to cobalt and zinc, were subjected to two-way ANOVA. The data on experimental repetitions were pooled when no significant differences were found according to the *F*-test (α > 0.05). In the case of proteolytic activity, antibacterial activity, and resistance to cobalt and zinc, the data were subsequently analyzed using one-way ANOVA, and mean comparisons were performed with Tukey's test (α = 0.05). Student's *T*-test (α = 0.05) was used as a *post hoc* test for mean comparison in the case of antifungal activity. All these statistical tests were carried out using Statistica 7.0 (StatSoft, USA).

## Results

### Genome assembly and sequence comparison

**Figure 1:**
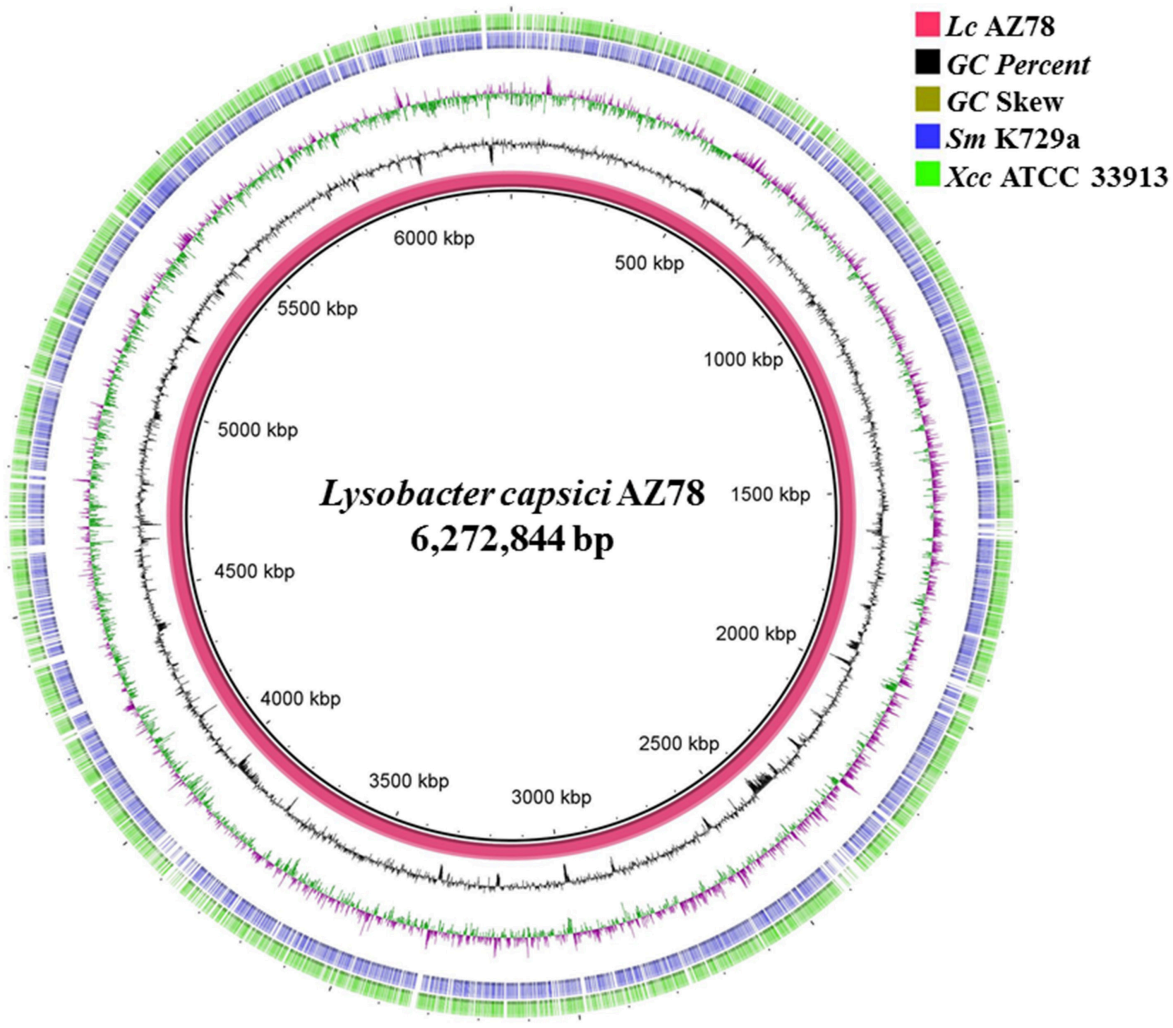
**Graphic representation of the *Lysobacter capsici* AZ78 genome**. Circles show (from the inside): (1) *Lc* AZ78 genome; (2) GC percent; (3) GC skew (4,5); Blast comparison of the *Lc* AZ78 genome with *Sm* K729a and *Xcc* ATCC 33913.

The PacBio SMRT cell yielded output data with average genome coverage of ~44x to generate a *de novo* assembly of the complete genome sequence of *Lc* AZ78. The PacBio RSII sequencing system generated 298,751,307 base pairs (bp) through 67,899 reads (N50 read length 6848 and mean read length 4399). The genome of *Lc* AZ78 (Figure 1) consists of 6,272,844 bp assembled into three contigs, and the G+C content is 66.4% (Table 1), similarly to the *L. capsici* type strain (65.4%; Park et al., 2008). The *Lc* AZ78 genome contains 5292 predicted coding sequences and 93 predicted non-coding RNAs, including one transfer-messenger RNA (tmRNA), seven rRNAs, and 85 tRNAs (Table 1).

**Table 1:** **Summary of the genomic characteristics of *Lysobacter capsici* AZ78, *Stenotrophomonas malthophilia* K279a and *Xanthomonas campestris* pv. *campestris* ATCC 33913**.

	***Lysobacter capsici* AZ78**	***Stenotrophomonas malthophilia* K729a**	***Xanthomonas campestris* pv. *campestris* ATCC 33913**
Contigs	3	1	1
Number of bases (bp)	6,272,844	4,851,126	5,076,187
G+C content (%)	66.43	66.70	65.00
Number of predicted coding sequences	5292	4386	4182
Coding percentage	82.8	88.8	84.34
rRNA	7	4	2
tRNA	85	74	53

**Figure 2:**
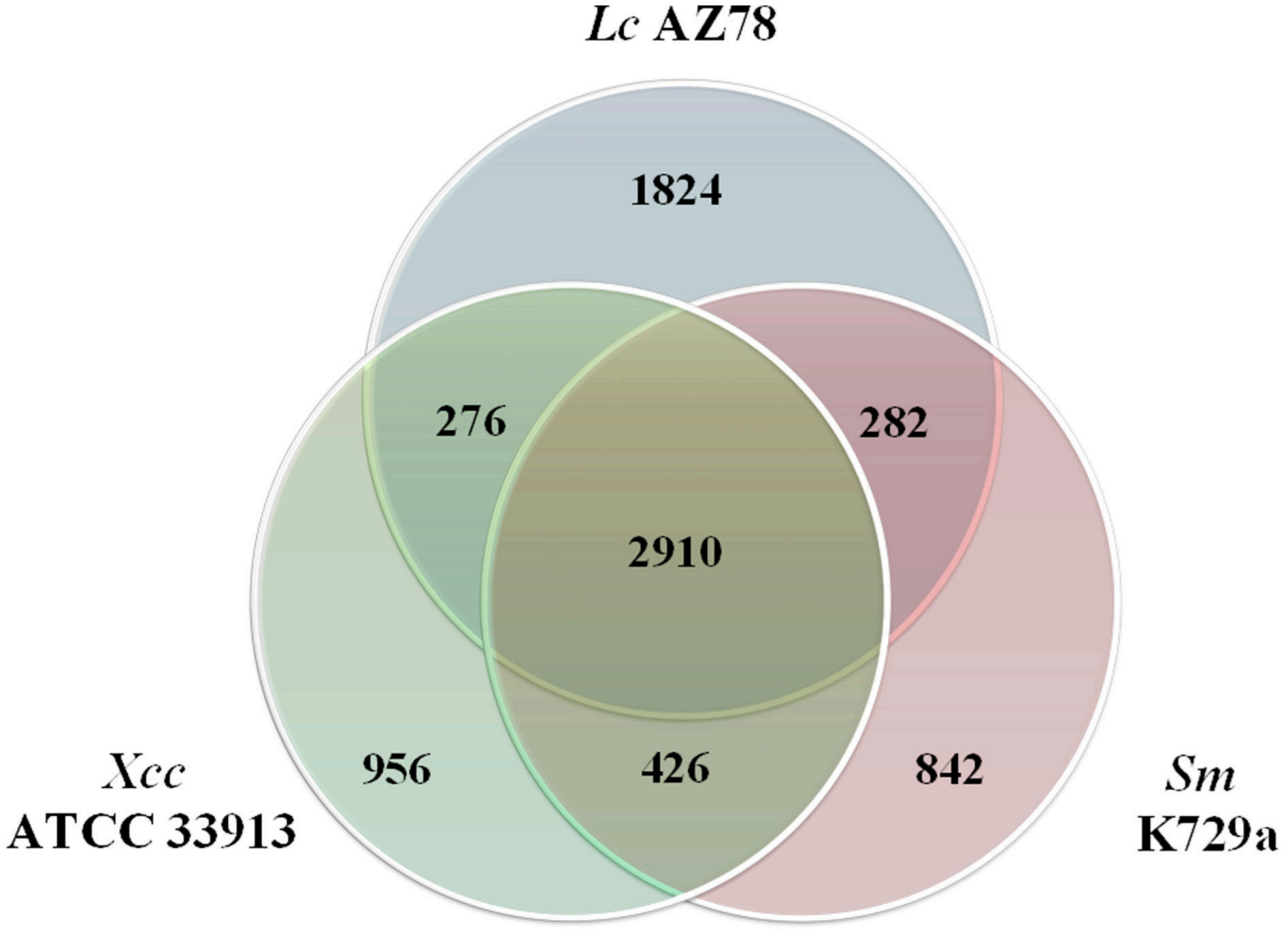
**Comparison between the genomes of *Lysobacter capsici* AZ78, *Stenotrophomonas malthophilia* K729a and *Xanthomonas campestris* pv. *campestris* ATCC 33913**. The Venn Diagram shows the number of shared and genome-specific genes in *Lc* AZ78, *Sm* K729a and *Xcc* ATCC 33913.

The *Lc* AZ78 genome was compared with the genomes of the opportunistic human pathogen *Sm* K729a and the phytopathogen *Xcc* ATCC 33913 (da Silva et al., 2002; Crossman et al., 2008). Genome characteristics such as chromosome size, G+C content and the number of predicted coding sequences differed in the three bacterial strains (Table 1). They share a core genome of 2910 orthologs, mostly involved in primary metabolism (Figure 2). Genes that are associated with the capacity of *Sm* K729a to infect humans, such as the *smlt0598, smlt3048, smlt4452*, and *wbpv* genes (Crossman et al., 2008), are absent in the genome of *Lc* AZ78 and *Xcc* ATCC 33913. Similarly, the *avrBs1, avrBs1.1, avrBs2, avrXccA1, avrXccA2, avrXccB* and *avrXccC* genes involved in the pathogenic interaction (da Silva et al., 2002; Wang et al., 2007) are specific for the *Xcc* ATCC 33913. The *Lc* AZ78 genome contains several unique genes involved in interaction processes with other microorganisms and environmental factors (Table 2).

**Table 2:** **List of genes specific to *Lysobacter capsici* AZ78 or shared with *Stenotrophomonas malthophilia* K279a and *Xanthomonas campestris* pv. *campestris* ATCC 33913**.

	**Shared features**		**Specific features**	
	**Name**	**Biological activity**	**Name**	**Biological activity**
Interaction with microorganisms	*expR* (AZ78_4489, 4492, 4498, 4503, 4505)	Production of extracellular protease	Proteolytic Region (AZ78_4508, 4509, 4511, 4512)	Production of zinc extracellular proteases
	*gluA* (AZ78_3675)	Degradation of glucans	Proteolytic Region (AZ78_4514, 4516)	Production of bacterial leucyl aminopeptidase
	*chiA* (AZ78_1828)	Degradation of chitin	Metalloendopeptidase Region (AZ78_269, 271, 272)	Production of metalloendopeptidases
	*feoABC* (AZ78_5035-5037)	Uptake of ferrous iron	*cel5G* (AZ78_3681)	Degradation of cellulose
			*gluB* (AZ78_4722); *gluC* (AZ78_1531); KF738079 (AZ78_4157*)*; *cel8A* (AZ78_4006); *celA_1_* (AZ78_4352)	Degradation of glucans
			*chiB* (AZ78_54)	Degradation of chitin
			NPR-PKS (AZ78_1098)	Production of antifungal compounds
			*lanL* (AZ78_848)	Production of lantibiotics
			*csbC-entEBF-viuB-entA* (AZ78_407-412)	Synthesis of cathecol siderophores
Interaction with environment	*pigABCDEFG* (AZ78_3467-3472)	Biosynthesis of xanthomonadin	Catalase/Peroxidase (AZ78_681, 1116, 1469)	Resistance to reactive oxygen species
	*copA, copB* (AZ78_402-403)	Resistance to copper ions	*cphA-cphB* (AZ78_4099-4100)	Cyanophycin metabolism
	*czcCBA* (AZ78_3809-3811)	Resistance to heavy metals	Copper efflux region (AZ78_560-562)	Resistance to copper ions
	Resistance-Nodulation-Division (AZ78_451, 452, 826)	Resistance to toxic compounds	SMR protein (AZ78_906, 1192, 2446)	Resistance to toxic compounds
	ABC transporters (AZ78_929, 967, 3014)	Resistance to β-lactams	MFS protein (AZ78_266, 1103, 3068, 3698, 3949, 4767)	Resistance to toxic compounds
	*blaL2* (AZ78_ 3946)	Resistance to β-lactams	Kanamycin nucleotidyltransferase (AZ78_3393)	Resistance to aminoglycosides
			B-lactamases (AZ78_238, 2665, 3448, 3627, 4028)	Resistance to β-lactams

### Interaction with microorganisms: production of lytic enzymes

The presence in *Lc* AZ78 genome of 79 genes encoding proteolytic enzymes represents a substantial difference with *Sm* K729a and *Xcc* ATCC 33913 genomes. A region of 40,829 bp in length is specific to the *Lc* AZ78 genome and is missing from the genome of the other two bacterial species (Proteolytic Region, Table 2). Although this region contains five genes (AZ78_4489, 4492, 4498, 4503, and 4505) encoding extracellular proteases homologous to an extracellular protease present in the genome of *Sm* K729a (*expR*, Smlt0861; Table 2) and *Xcc* ATCC 33913 (XCC0851), it has other *Lc* AZ78-specific genes. In particular, four genes (AZ78_4508, 4509, 4511, and 4512) encoding extracellular zinc proteases (EC 3.4.24.26), and two genes (AZ78_4514 and 4516) encoding bacterial leucyl aminopeptidase (EC 3.4.11.10) have no orthologs in the *Sm* K729a and *Xcc* ATCC 33913 genomes. Another *Lc* AZ78-specific region (Metalloendopeptidase Region, Table 2) contains three *Lc* AZ78-specific genes (AZ78_269, 271 and 272) encoding metalloendopeptidases that do not share homology with any gene of *Sm* K729a and *Xcc* ATCC 33913. However, peptidase genes that have already been characterized in other *Lysobacter* strains were found in *Lc* AZ78, such as three genes encoding endopeptidases and a peptidyl Asp-metalloendopeptidase homologous to the *lepA* (AB045676) and *lepB* (AB094439) genes of *Lysobacter* sp. IB-9374, respectively (Chohnan et al., 2002, 2004).

**Figure 3:**
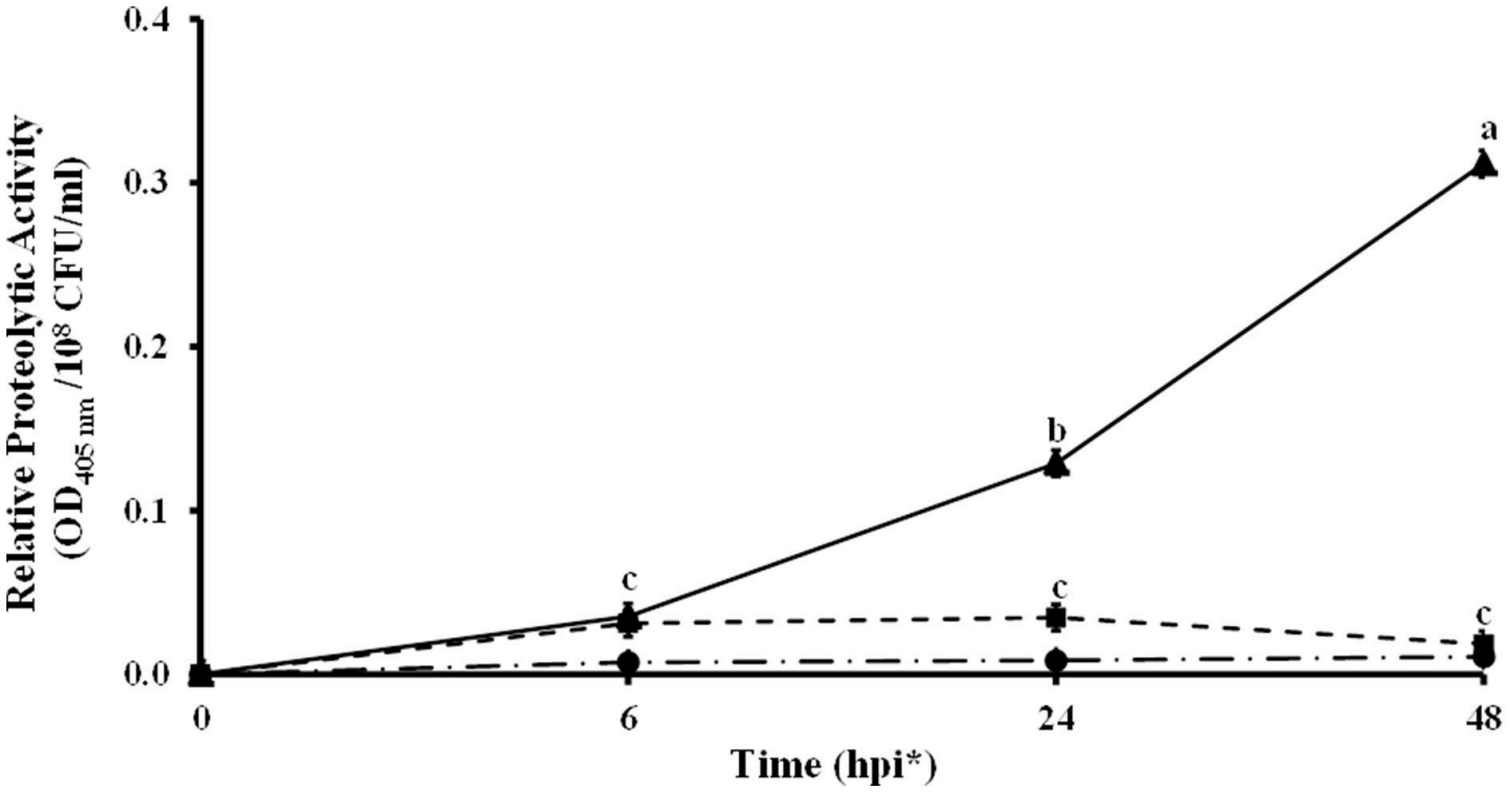
**Proteolytic activity in the *in vitro* interaction between *Lysobacter capsici* AZ78 and *Phytophthora infestans***. Proteolytic activity was monitored 6, 24, and 48 h after the following treatments: *Lc* AZ78 (●), *P. infestans* (■) and *Lc* AZ78 + *P. infestans* (▲). Mean and standard error values for six replicates (2 ml-tubes) pooled from two experiments are reported for each condition. Different letters indicate significant differences according to Tukey's test (α = 0.05).

Other genes located in these two regions share homology with protease genes (KF738078, KF738082, KF738069, and KF738070) of *L. gummosus* UASM 402 (Gökçen et al., 2014). The high number of proteases found in *Lc* AZ78 genome, led to investigate whether proteolytic activity could be involved in the interaction between *Lc* AZ78 and the phytopathogen *P. infestans*. A significant increase in proteolytic activity occurred when the two microorganisms were co-cultured at 25°C for 24 h (Figure 3).

**Figure 4:**
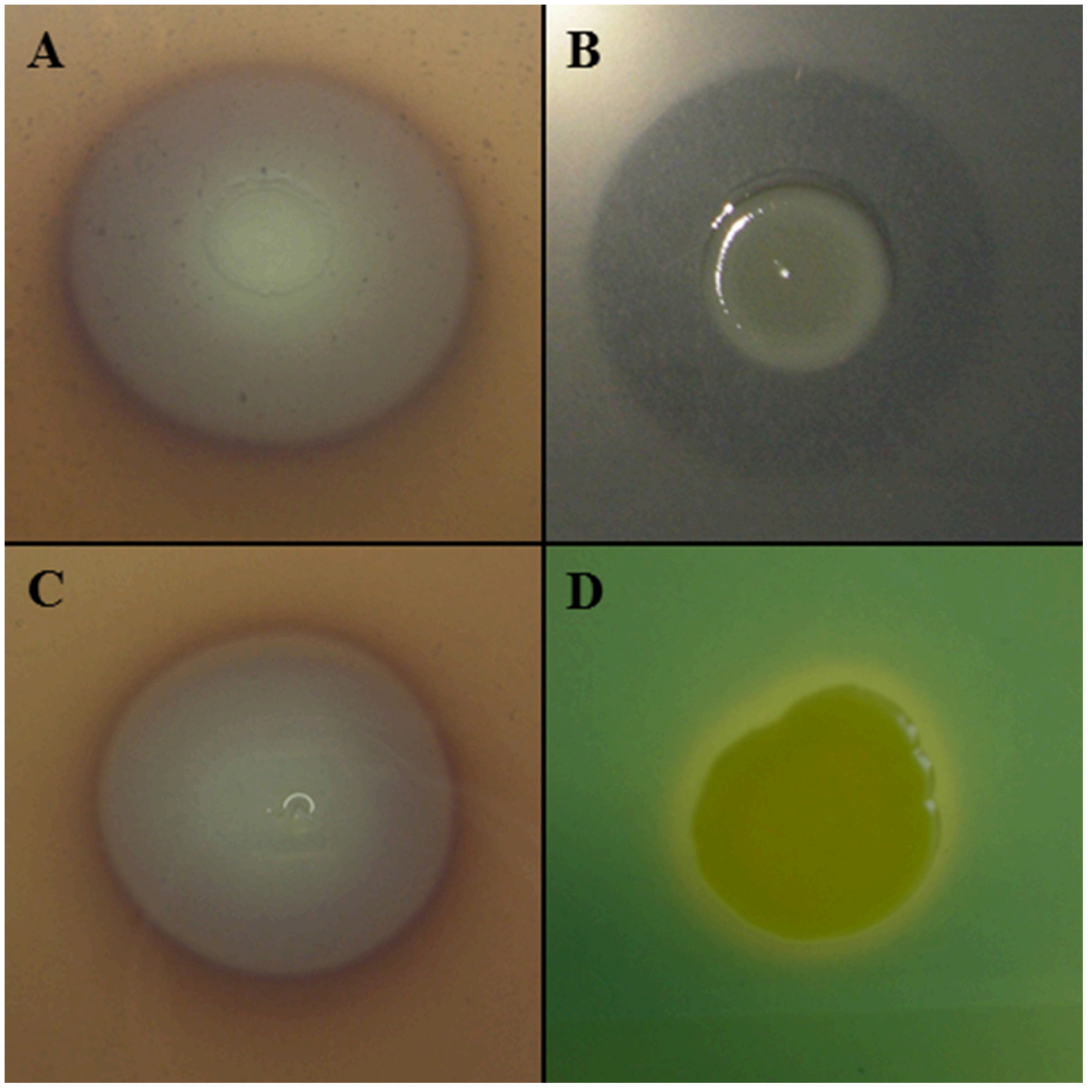
**Characterization of *Lysobacter capsici* AZ78**. *Lc* AZ78 produces **(A)** cellulases; **(B)** β-glucanases; **(C)** chitinases, and **(D)** siderophores.

Other lytic enzymes of *Lc* AZ78 may be involved in the degradation of other components of *P. infestans* cell wall such as cellulose that was degraded *in vitro* by *Lc* AZ78 (Figure 4A). Cellulose degradation is related to the presence in *Lc* AZ78 genome of the AZ78_3681 gene encoding a cellulase belonging to glycosyl hydrolase family 5 that has no homology with cellulases included *Sm* K729a and *Xcc* ATCC 33913 genomes (Table 2). However, cellulase from *Lc* AZ78 shows homology at amino acid level with the Cel5G, a putative cellulase found in the genome of *Cellvibrio japonicus* Ueda107 (CP000934; DeBoy et al., 2008).

*L. capsici* AZ78 was able to degrade laminarin *in vitro* (Figure 4B) and this lytic activity was confirmed by the presence in its genome of three genes encoding two enzymes (GluA, GluC) belonging to glycosyl hydrolase family 16, and one enzyme (GluB) belonging to glycosyl hydrolase family 64. Specifically, three genes (AZ78_3675, 4722, 1531) are orthologs of *gluA, gluB* and *gluC* previously characterized in *L. enzymogenes* C3 (AY667477; AY667478; AY667479) and N4-7 (AY157838; AY157839; AY157840; Palumbo et al., 2003, 2005). The *Sm* K729a genome lacks genes encoding endo β-1,3 glucanases, while *Xcc* ATCC 33913 has a gene (XCC1188) encoding an endo β-1,3 glucanase homologous to *gluA* of *Lc* AZ78 (Table 2). Unlike *L. enzymogenes* C3, N4-7 and *Xcc* ATCC 33913, *Lc* AZ78 has a second gene (AZ78_406) encoding an endo β-1,3 glucanase homologous to GluA, a gene (AZ78_4157) encoding an enzyme belonging to glycosyl hydrolase family 16 showing considerable similarity with the KF738079 gene identified in *L. gummosus* UASM 402 (Gökçen et al., 2014), and another gene (AZ78_4006; Table 2) which is homologous to *cel8A* of *Lysobacter* sp. IB-9374 (AB244037) encoding an enzyme belonging to glycosyl hydrolase family 8 with β-1,4 glucanase and chitosanase activity (Ogura et al., 2006). *L. capsici* AZ78 also has a gene (AZ78_4352) that encodes a β-1,4 endoglucanase belonging to glycosyl hydrolase family 6, which has never been characterized to date in *Lysobacter* members. This is missing in the genome of *Sm* K729a and *Xcc* ATCC 33913 (Table 2), although it is homologous to *celA*_1_ (Z12157) described for *Streptomyces halstedii* JM8 (Fernández-Abalos et al., 1992).

As regards phytopathogenic fungi, *Lc* AZ78 degraded chitin *in vitro* (Figure 4C), and this activity is related to the presence in its genome of the AZ78_1828 gene encoding a chitinase A present in the *Sm* K729a genome (Smlt0682, Table 2) and not in the *Xcc* ATCC 33913 genome. Unlike *Sm* K729a, the *Lc* AZ78 genome contains another gene (AZ78_3859) encoding a chitinase A that shares high similarity with the *chiA* gene (AB014770) described in *Xanthomonas* sp. AK (Sakka et al., 1998). *L. capsici* AZ78 genome also has a gene (AZ78_54) encoding a chitinase B (*chiB*, Table 2) sharing homology with *chiB* genes identified in the two strains *Burkholderia gladioli* CHB101 (AB038998) and BSR3 (CP002600; Shimosaka et al., 2001; Seo et al., 2011).

### Interaction with microorganisms: production of antibiotics

*L. capsici* AZ78 released secondary metabolites with antifungal activity *in vitro* and reduced the in *vitro* mycelial growth of 22 phytopathogenic fungi, with the sole exception of *Pyrenochaeta (Py.) lycopersici* (Table 3). *L. capsici* AZ78 genome was mined for genes potentially involved in the production of antibiotics and a genomic region of 9489 bp missing in *Sm* K729a and *Xcc* ATCC 33913 was identified. Specifically, the AZ78_1098 gene (Table 2) has homology with a gene encoding a hybrid polyketide synthase and a non-ribosomal peptide synthetase (NPR-PKS) involved in the biosynthesis of Heat Stable Antifungal Factor (HSAF) in *L. enzymogenes* C3 (EF028635; Yu et al., 2007).

**Table 3:** **Antifungal activity of *Lysobacter capsici* AZ78**.

**Phytopathogenic fungus**	**Untreated**	***Lc* AZ78**
*Alternaria alternata*	4.50 ± 0.05	1.50 ± 0.16^*^
*Ascochyta rabiei*	6.10 ± 0.12	1.36 ± 0.06^*^
*Aspergillus flavus*	2.32 ± 0.07	0.65 ± 0.03^*^
*Aspergillus niger*	2.50 ± 0.02	0.58 ± 0.02^*^
*Aspergillus ochraceus*	2.68 ± 0.09	0.48 ± 0.04^*^
*Botrytis cinerea*	7.45 ± 0.10	2.54 ± 0.12^*^
*Colletotrichum gloeosporioides*	7.86 ± 0.21	1.84 ± 0.14^*^
*Fusarium acuminatum*	7.92 ± 0.12	3.72 ± 0.16^*^
*Fusarium avenaceum*	3.56 ± 0.13	1.77 ± 0.24^*^
*Fusarium oxysporum* f. sp. *asparagi*	7.56 ± 0.34	4.34 ± 0.18^*^
*Fusarium oxysporum* f. sp. *lycopersici*	7.34 ± 0.25	3.10 ± 0.15^*^
*Fusarium oxysporum* f. sp. *radicis-lycopersici*	7.12 ± 0.21	3.08 ± 0.04^*^
*Fusarium sambucinum*	7.11 ± 0.12	2.24 ± 0.08^*^
*Fusarium semitectum*	7.83 ± 0.08	4.42 ± 0.12^*^
*Fusarium solani*	7.95 ± 0.09	2.66 ± 0.11^*^
*Penicillium* sp.	3.52 ± 0.20	1.76 ± 0.06^*^
*Phoma tracheiphila*	3.92 ± 0.21	2.23 ± 0.06^*^
*Pyrenochaeta lycopersici*	7.84 ± 0.08	7.73 ± 0.13^*^
*Rhizoctonia solani*	7.89 ± 0.11	1.73 ± 0.16^*^
*Sclerotinia maior*	7.86 ± 0.14	1.84 ± 0.08^*^
*Sclerotinia minor*	7.92 ± 0.15	1.86 ± 0.20^*^
*Sclerotinia sclerotiorum*	7.94 ± 0.13	1.46 ± 0.27^*^
*Thielaviopsis basicola*	7.89 ± 0.17	3.50 ± 0.16^*^

*The antifungal activity of Lc AZ78 was assessed through dual-culture assay. Mean and standard errors values of mycelial growth diameters (cm) with six replicates (Petri dishes) pooled from two experiments are reported for each fungus*.

* *Values followed by asterisks differ significantly according to Student's t-test (α = 005)*.

In our antibacterial activity tests, *Lc* AZ78 released compounds that are toxic to the Gram-positive phytopathogenic bacteria *Clavibacter michiganensis* subsp. *michiganensis* LMG 7333, *C. michiganensis* subsp. *sepedonicus* LMG 2889, *Rhodococcus fascians* LMG 3605 and *Streptomyces turgidiscabies* DSM 41997, while no toxic activity was shown against the tested Gram-negative phytopathogenic bacteria (Table 4). *L. capsici* AZ78 genome hosts genes involved in the biosynthesis of ribosomally encoded antibacterial peptides named lantibiotics (Chatterjee et al., 2005), and these genes are missing in the genomes of *Sm* K729a and *Xcc* ATCC 33913 (Table 2). *L. capsici* AZ78 contains a gene (AZ78_848) homologous to the *venL* gene (WP_015031826) identified in *Streptomyces venezuelae* ATCC 10712 (Goto et al., 2010). As for *venL* in *S. venezuelae* ATCC 10712, the AZ78_848 gene is followed by two genes (AZ78_847 and AZ78_846) encoding an ATP-binding protein and an efflux transporter, indicating synteny of this region in the two bacterial species.

**Table 4:** **Antibacterial activity of *Lysobacter capsici* AZ78**.

**Phytopathogenic bacterium**	**Halo of inhibition zone (mm)^a^**
*Agrobacterium tumefaciens*	0 ± 0
*Erwinia carotovora* subsp. *carotovora*	0 ± 0
*Ralstonia solanacearum*	0 ± 0
*Xanthomonas campestris* pv. *campestris* DSM 3586	0 ± 0
*Clavibacter michiganensis* subsp. *michiganensis* LMG 3690	12.44 ± 0.55^a^
*Clavibacter michiganensis* subsp. *sepedonicus* LMG 3690	12.77 ± 0.16^a^
*Rhodococcus fascians* LMG 3605	10.38 ± 1.06^a^
*Streptomyces turgidiscabies* DSM 41838	11.05 ± 0.65^a^

a *The production of secondary metabolites with antibacterial activity by Lc AZ78 was assessed against Gram-negative and Gram-positive phytopathogenic bacteria. Antibacterial activity is expressed as the mean value of the halo inhibition zone ± standard errors. Six replicates (Petri dishes) pooled from two experiments are reported for each bacterium. No significant differences were present in inhibition zones according to Tukey's test (α = 005)*.

### Interaction with microorganisms: production of siderophores

*L. capsici* AZ78 produced siderophores on CAS agar plates (Figure 4D) and has genes involved in uptake and transport of iron ions that are homologous to genes present in the genome of *Sm* K729a and *Xcc* ATCC 33913. For example, the *Lc* AZ78 genome includes the *feoABC* operon (AZ78_5035-5037, Table 2) involved in the uptake of ferrous iron, which shares high homology with the *feoABC* operon present in *Sm* K729a (Smlt2211-2213), *Xcc* ATCC 33913 (XCC1834-1836). *L. capsici* AZ78 contains an additional gene cluster (AZ78_407-AZ78_412, Table 2) whose genes show homology with the *entAFBE-csbC* (CP001157) genes responsible for the production of catechol siderophores in *Azotobacter vinelandii* strains DJ and ATCC 12837 (Setubal et al., 2009; Yoneyama et al., 2011). The corresponding region in *Lc* AZ78 contains one gene (AZ78_411) encoding a protein similar to ViuB (CP001235), involved in the utilization of exogenous ferric vibriobactin complex in *Vibrio cholerae* 0395 (Butterton and Calderwood, 1994).

### Interaction with the environment: tolerance to environmental stressors

The genetic information needed for resistance to environmental stressors is shared by the *Lc* AZ78, *Sm* K729a and *Xcc* ATCC 33913 genomes. For instance, *Lc* AZ78 genome has genes involved in the biosynthesis of xanthomonadin (AZ78_ 3467-3472, Table 2), a pigment responsible for protection against UV light irradiation in *Xanthomonas* spp. (Rajagopal et al., 1997). Moreover, *Lc* AZ78 includes several genes related to reactive oxygen species (ROS) resistance and shared with *Sm* K729a and *Xcc* ATCC 33913 and genes encoding a cytochrome c551 peroxidase (EC 1.11.1.5) (AZ78_681), a catalase (EC 1.11.1.6)/Peroxidase (EC 1.11.1.7) (AZ78_1116) and a superoxide dismutase (EC 1.15.1.1) (AZ78_1469) with no orthologs in the *Sm* K729a and *Xcc* ATCC 33913 genomes (Table 2).

Another specificity of *Lc* AZ78 genome is represented by a two-gene cluster (AZ78_4099-4100) responsible for the biosynthesis and degradation of cyanophycin with no orthologs in *Sm* K729a and *Xcc* ATCC 33913 (Table 2). The synthesis and degradation of cyanophycin are catalyzed respectively by cyanophycin synthetase and cyanophycinase encoded by the two-gene cluster *cphA-cphB* (Li et al., 2001; Krehenbrink et al., 2002). *L. capsici* AZ78 genome hosts a cyanophycinase (AZ78_4099), followed immediately by a cyanophycyn synthetase (AZ78_4100).

### Interaction with the environment: tolerance to heavy metals

The *Lc* AZ78, *Sm* K729a, and *Xcc* ATCC 33913 genomes have the *copA* and *copB* genes, encoding a multicopper oxidase and the copper resistance protein B respectively (AZ78_402-403, Table 2). Unlike the other two Xandomonadaceae members, *Lc* AZ78 has a genomic region encoding proteins involved in the efflux of copper ions (Table 2). This region contains two Cu^2+^ exporting ATPases (EC 3.6.3.4; AZ78_560-561), homologous with CP002600 of *B. gladioli* BSR3 (Seo et al., 2011). The product of the gene AZ78_562, located downstream of the AZ78_560-561 genes, belongs to the MerR superfamily of transcriptional activators (Hobman and Brown, 1997) and is homologous to a Cu(I)-responsive transcriptional activator (*cueR*) of the copper efflux system in γ-proteobacteria (Stoyanov et al., 2001).

**Figure 5:**
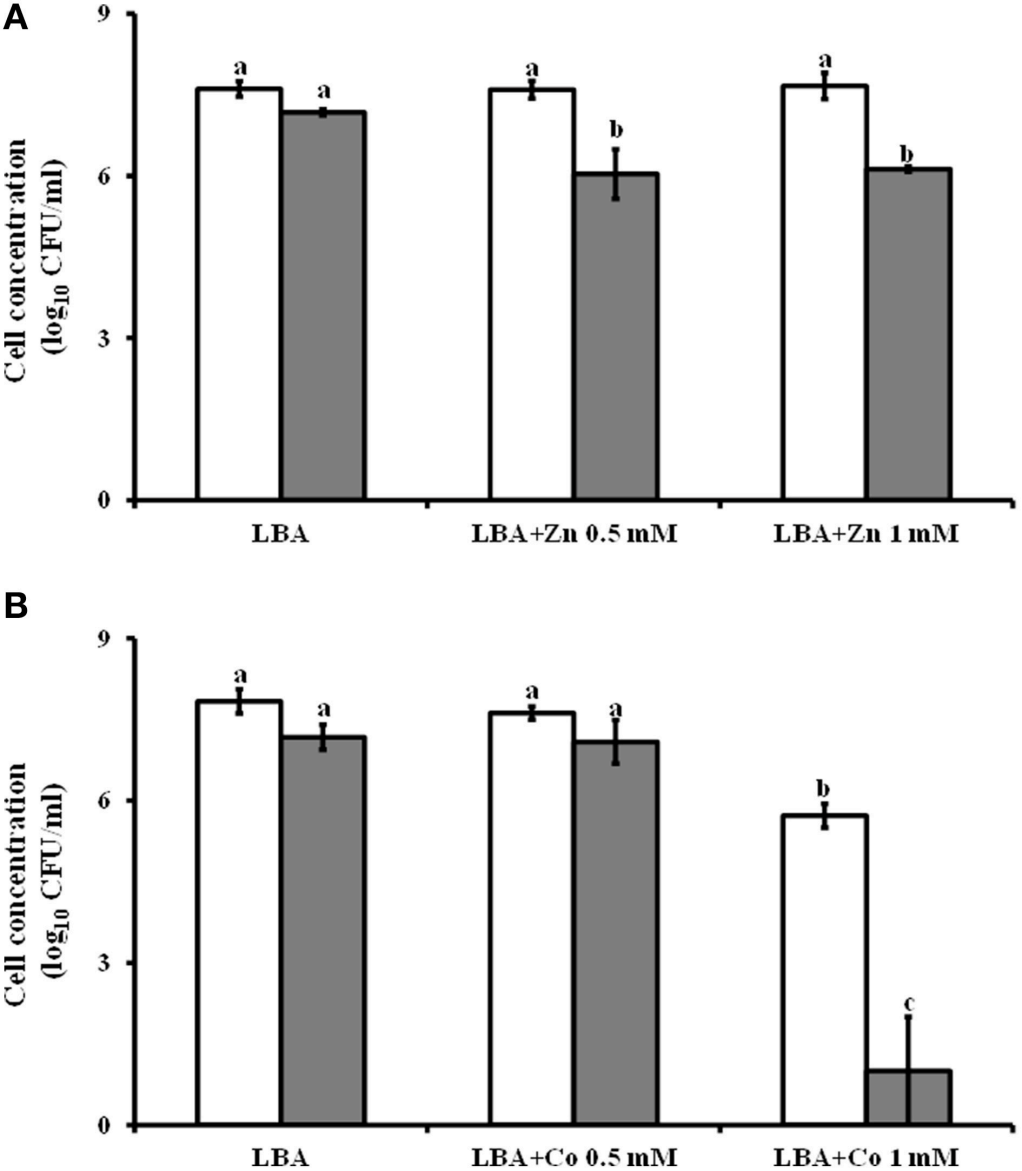
**Assessment of *Lysobacter capsici* AZ78 resistance to heavy metals**. *Lc* AZ78 (white columns) was evaluated to assess its ability to resist zinc **(A)** and cobalt **(B)** at two concentrations (0.5 and 1 mM). *B. amyloliquefaciens* FZB42 (gray columns) was used as control. Mean and standard error values (columns) for six replicates (Petri dishes) pooled from two experiments are reported for each heavy metal concentration. Different letters indicate significant differences according to Tukey's test (α = 0.05).

The *Lc* AZ78 genome has a 4396 bp region containing three genes (AZ78_3809-3811) that are homologous with the *czcCBA* operon involved in resistance to cadmium, cobalt and zinc (Nies, 2003). This operon (Table 2) is also present in the genomes of *Sm* K729a (Smlt2456-2458) and *Xcc* ATCC 33913 (XCC4036-4038). The *Lc* AZ78 genome also hosts a gene (AZ78_3808) encoding an ortholog of the CzcD protein involved in the expression regulation of *czcCBA* in *Ralstonia* sp. CH34 (Anton et al., 1999). The presence of these genes was associated with the ability of *Lc* AZ78 to grow on LBA amended with different concentrations of cobalt and zinc (Figure 5). In this *in vitro* experiments, *B. amyloliquefaciens* FZB42 was used as control since its genome is missing of *czcCBA* operon (CP000560; Chen et al., 2007). The viability of *Lc* AZ78 was not negatively affected by ZnSO_4_ at the concentrations tested while *B. amyloliquefaciens* FZB42 viability was reduced of an order of magnitude at both the concentrations tested (Figure 5A). CoCl_2_ resulted more toxic against *Lc* AZ78 cells and a decrease of two orders of magnitude was registered on LBA amended with 1 mM CoCl_2_ (Figure 5B). However, a more drastic reduction in *B. amyloliquefaciens* FZB42 was observed at the same conditions (Figure 5B).

### Interaction with the environment: tolerance to fungicides and antibiotics

**Figure 6:**
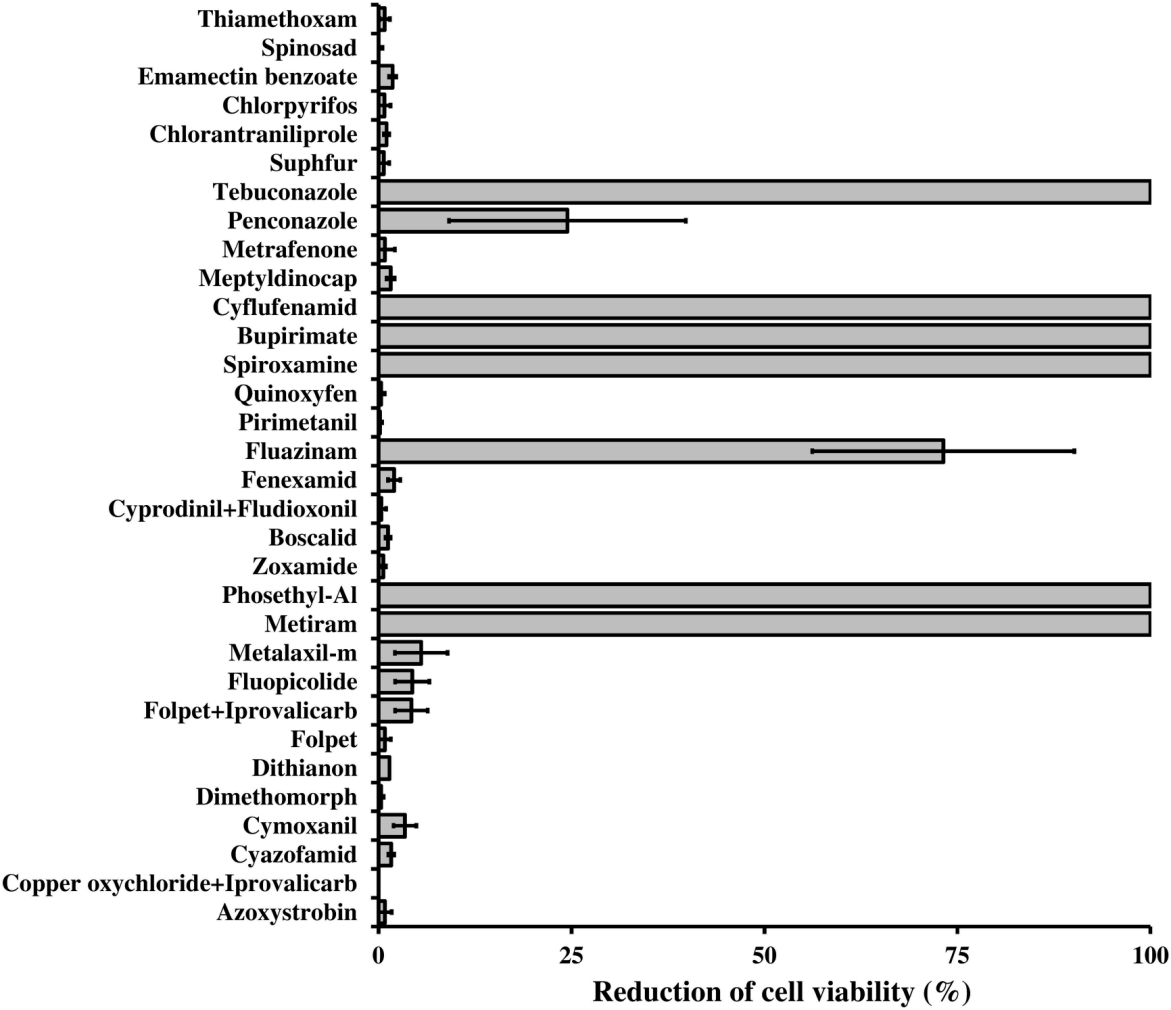
**Resistance of *Lysobacter capsici* AZ78 to plant protection products**. The survival of *Lc* AZ78 cells in the presence of fungicides and insecticides was assessed by growing the bacterium on LBA amended with plant protection products at concentrations commonly applied in the field. The reduction in cell viability was calculated as the ratio between the CFU difference for *Lc* AZ78 grown on LBA and *Lc* AZ78 grown on LBA amended with plant protection products, over *Lc* AZ78 CFU grown on LBA. Mean and standard error values (columns) for six replicates (Petri dishes) pooled from two experiments are reported for each plant protection product.

*L. capsici* AZ78 was resistant *in vitro* to several fungicides and insecticides commonly applied in viticulture (Figure 6). This resistance may rely on the presence of efflux systems such as ABC transporters, Resistance-Nodulation-Division (RND), Small Multidrug Resistance protein (SMR), and proteins belonging to the Major Facilitator Super-family (MFS) and the Multidrug Toxic compound Extrusion family (MTE; Poole, 2001), which *Lc* AZ78 shares with *Sm* K729a and *Xcc* ATCC 33913 (Table 2). Moreover, there are some *Lc* AZ78-specific genes related to fungicide and antibiotic resistance, such as three genes (AZ78_906, 1192, and 2446) encoding putative SMR proteins and six genes (AZ78_266, 1103, 3068, 3688, 3949, and 4767) encoding putative MFS proteins (Table 2).

Since all these efflux systems can also contribute to resistance to antibiotics, we evaluated the sensitivity of *Lc* AZ78 to several antibiotics *in vitro* (Table 5). *L. capsici* AZ78 is sensitive to chloramphenicol, erythromycin, gentamicin, tetracycline, trimethoprim and vancomycin whereas it is resistant to ampicillin, kanamycin, streptomycin, and tobramycin. The resistance to kanamycin and streptomycin may depend on inactivation mediated by aminoglycoside phosphotransferase or adenylyltransferase enzymes (Shaw et al., 1993). The *Lc* AZ78 genome contains a gene (AZ78_3072) encoding putative streptomycin 3″-kinase (EC 2.7.1.87) that does not show homology with *aph*(3′′) and *aph*(3′)*-IIc* involved in streptomycin and kanamycin resistance in *Sm* K729a (Okazaki and Avison, 2007; Crossman et al., 2008). Moreover, the *Lc* AZ78 genome includes another gene (AZ78_3393) encoding a putative kanamycin nucleotidyltransferase (Table 2). Other putative antibiotic resistance genes in *Lc* AZ78 genome are responsible for resistance to β-lactams such as ampicillin: 12 genes showed homology with the β-lactamases of *Sm* K729a and *Xcc* ATCC 33913 and five β-lactamases genes (AZ78_238, 2665, 3488, 3627, and 4028) were unique to *Lc* AZ78 (Table 2).

**Table 5:** **Assessment of *Lysobacter capsici* AZ78 resistance to antibiotics**.

**Antibiotic**	**Disc content (μg)**	**Diameter of inhibition zone (mm)**	**Zone diameter breakpoint^a^ (mm)**
Ampicillin	10	0.00±0.00	<14
Chloramphenicol	30	52.10±1.01	<19
Erythromycin	30	45.83±0.81	
Gentamicin	30	23.33±0.21	<17
Kanamycin	30	0.00±0.00	
Streptomycin	25	0.00±0.00	<12
Tetracycline	30	43.90±0.55	<17
Tobramycin	10	0.00±0.00	<16
Trimethoprim	5	23.23±0.67	<13
Vancomycin	30	23.70±0.21	

*Lc AZ78 was evaluated for its resistance to different antibiotics. The results are the mean diameters ± standard errors of pooled data for a repeated experiment with three replicates of each antibiotic for each experiment. The diameters of inhibition zone were determined to assign resistance or sensitivity to each antibiotic*.

a *Cut-off diameters to define resistance to the tested antibiotic (Andrews et al., 2011; Anonymous, 2011)*.

## Discussion

Development of new biofungicides based on BCAs are becoming an important task to reduce the use of chemical fungicides for the control of phytopathogenic microorganisms. Recently, *Lc* AZ78 was shown to effectively control two important phytopathogenic oomycetes and a first formulation prototype was designed for its application in vineyards for the control of *Pl. viticola* (Puopolo et al., 2014a,b; Segarra et al., 2015a,b). However, information regarding the biological characteristics of *Lc* AZ78 are needed for the registration process and to foster the development of this bacterial strain as the main ingredient of novel biofungicides. Thus, the genome of *Lc* AZ78 was sequenced by PacBio sequencing system. This technology was chosen because the advantage of longer reads during assembly ensures a higher quality final result (Roberts et al., 2013). The drawback of higher error rate for PacBio sequences can be overcome with a higher genome coverage, a choice that already proved to be the best strategy nowadays for bacterial sequencing projects (Booher et al., 2015; Cameron et al., 2015; Lee et al., 2015). Accordingly, PacBio RSII sequencing system resulted more effective than the Illumina GAIIx system previously used for obtaining a first draft genome of *Lc* AZ78 (Puopolo et al., 2014c) and number of contigs was reduced from 142 achieved with Illumina to three achieved with PacBio in this work.

Since comparison of the genomes highlighted important similarities and differences between microorganisms (Studholme et al., 2009; Straub et al., 2013), the *Lc* AZ78 genome was compared with the genomes of the opportunistic human pathogen *Sm* K729a and the phytopathogen *Xcc* ATCC 33913 originally sequenced using Sanger sequencing technology (da Silva et al., 2002; Crossman et al., 2008). This technology is no longer used for whole genome assembly projects, however the quality of Sanger sequencing technology is comparable to the quality achieved with PacBio technology (Eid et al., 2009). The comparative analysis of the three genomes revealed high diversity between the three bacterial strains and showed that *Lc* AZ78 does not have the genetic basis for pathogenic interaction with humans and plants. Particularly, the comparison highlights the lack in *Lc* AZ78 genome of genes responsible for the instauration of human diseases present in *Sm* K729a genome. Similarly, the lack of these genes represents one of the factors differentiating the opportunistic human pathogen *Sm* K729a from the closely related beneficial bacterium *Stenotrophomonas rhizophila* DSM14405^T^ (Alavi et al., 2014). This comparative analysis also allowed to identify several *Lc* AZ78-specific genes that are related to bacterial responses to other microorganisms and environmental factors.

A vast number of *Lc* AZ78-specific genes are involved in competition with other microorganisms highlighting the biocontrol properties of *Lc* AZ78 associated with the release of extracellular enzymes that can lyse the cell wall of both fungi and oomycetes. Members of the genus *Lysobacter* are known to produce a plethora of extracellular enzymes with lytic activity capable of degrading the cell wall components of several phytopathogenic microorganisms (Kobayashi and Yuen, 2007; Hayward et al., 2010). Accordingly, the *Lc* AZ78 genome includes a repertoire of genes encoding lytic enzymes capable of degrading cellulose, chitin, laminarin and proteins *in vitro*.

One of the main differences between *Lc* AZ78, *Sm* K729a and *Xcc* ATCC 33913 genomes, relies on the presence of a large number of genes encoding extracellular proteases in the *Lc* AZ78 genome that may display specific proteolytic activities. Particularly, *Lc* AZ78 genome has four gene sharing homology with proteases characterized in *L. gummosus* UASM 402, which are involved in the digestion of biofilm produced by *Staphylococcus epidermidis* (Gökçen et al., 2014). The presence of these genes in the genome of *Lc* AZ78 highlights the importance of members of the *L. capsici* species as potential new sources of enzymes exploitable for the control of important human pathogens through degradation of their extracellular matrix. Proteolitic activity of *Lc* AZ78 increased by *in vitro* incubation with *P. infestans*, and this may represent a key process in *Lc* AZ78′s biocontrol activity as shown for *L. enzymogenes* 3.1T8 (Folman et al., 2003, 2004). Furthermore, *Lc* AZ78 degraded *in vitro* β-glucans (laminarin) and cellulose other components of the oomycete cell wall. The degradation of these polymers is associated with the presence in the *Lc* AZ78 genome of genes encoding cellulases and a vast array of β-glucanases that clearly indicates that phytopathogenic oomycetes represent an optimal target of this BCA.

The *Lc* AZ78 genome also includes unique genes encoding chitinases that are commonly involved in the control of phytopathogenic fungi and they play a significant role in the biocontrol activity of *L. enzymogenes* C3 against *Bipolaris sorokiniana* (Zhang and Yuen, 2000). The *Lc* AZ78 genome has a gene encoding for a ChiA enzyme already characterized in the *L. enzymogenes* C3 (AY667480), N4-7 (AY667481) and OH11 (DQ888611) strains (Zhang et al., 2001; Sullivan et al., 2003; Qian et al., 2009). Moreover, the analysis of genes encoding chitinases in *Lc* AZ78 genome revealed the presence of a second gene with a high level of identity with the *chiA* gene previously characterized in *Xanthomonas* sp. AK (Sakka et al., 1998). This homology, and the 16S rDNA analysis carried out by Folman et al. (2003), represents strong evidence for misidentification in the case of *Xanthomonas* sp. AK, which in our opinion should be considered a *Lysobacter* sp. Analysis of the *Lc* AZ78 genome highlighted the presence of a gene encoding a chitinase B, a novelty for the genus *Lysobacter* and Xanthomonadaceae. Indeed, enzymes such as ChiB belong to glycosyl hydrolase family 19, which includes chitinases mostly identified in actinomycetes (Watanabe et al., 1999). Within Proteobacteria, *chiB* genes were only identified in the two strains *B. gladioli* CHB101 and BSR3 (Shimosaka et al., 2001; Seo et al., 2011). The presence of both ChiA and ChiB chitinases in the *Lc* AZ78 genome supports the potential of this bacterial strain to attack and degrade the cell wall of phytopathogenic fungi.

The genomic and functional information provided in this work demonstrates that *Lc* AZ78 has unique genes responsible for the synthesis of macrocyclic lactams toxic against phytopathogenic microorganisms. *L. enzymogenes* C3 produces HSAF a compound consisting of dihydromaltophilin and related macrocyclic lactams, toxic for several phytopathogenic fungi and oomycetes (Yu et al., 2007; Li et al., 2008). *Lysobacter* sp. SB-K88 synthesizes the macrocyclic lactams Xanthobaccin A, B and C, which are highly active *in vitro* against *Aphanomyces cochlioides* and *Pythium ultimum* (Nakayama et al., 1999). The *Lc* AZ78 genome includes regions involved in the production of macrocyclic lactams and the *in vitro* experiments let to hypothesize that the putative toxic compounds released by *Lc* AZ78 have probable similarities with xanthobaccins. Indeed, Nakayama et al. (1999) reported that *Py. lycopersici* was not sensitive to xanthobaccins produced *in vitro* by *Lysobacter* sp. SB SB-K88. Similarly, this phytopathogenic fungus was not sensitive to the toxic compounds released *in vitro* by *Lc* AZ78 in our experiments. Future work will be aimed at determining the chemical structure of the secondary metabolites with antifungal activity produced by *Lc* AZ78 to study the involvement of this class of antibiotics in the biological control of phytopathogenic fungi and oomycetes.

*L. capsici* AZ78 also released secondary metabolites toxic to four phytopathogenic Gram-positive bacteria and the presence of genes involved in their biosynthesis differed in *Lc* AZ78, *Sm* K729a and *Xcc* ATCC 33913 strains. Particularly, *Lc* AZ78 genome contains genes involved in the production of lantibiotics, compounds toxic to Gram-positive bacteria (Chatterjee et al., 2005) that are missing in the genome of the other two bacterial strains. Little is known about lantibiotic production in *Lysobacter* members, whereas it was reported that *L. enzymogenes* OH11 produces the cyclic lipodepsipeptide WAP-8294A2 active against the human pathogenic Gram-positive bacterium *Staphylococcus aureus* (Zhang et al., 2011). Therefore, we cannot rule out the possibility that the antibacterial activity of *Lc* AZ78 could be associated with the production of other toxic secondary metabolites, and chemical analysis is needed to further investigate this topic.

The comparison of the three genomes higlighted genetic informations regarding the ability of *Lc* AZ78 to scavenge ferrous ions from the environment through the production of siderophores (Neilands, 1995; Chu et al., 2010). Altough the production of these secondary metabolites is known to be important for human pathogenic, plant pathogenic and plant beneficial bacteria (Hamdan et al., 1991; Pandey and Sonti, 2010; Skaar, 2010), few information are available about siderophore production in *Lysobacter* spp. Differently from *Sm* K729a and *Xcc* ATCC 33913, the genome of *Lc* AZ78 is provided with the *entAFBE-csbC* operon responsible for the production of cathecol siderophores indicating that *Lc* AZ78 may also compete with other microorganisms for iron ions in the environment.

Our results also highlight key genes involved in the resistance of *Lc* AZ78 to UV-light irradiation and starvation. Previously, we have shown that *Lc* AZ78 resisted to starvation stress for 15 days and can be stored at 4°C in distilled water for a year (Puopolo et al., 2014a; Segarra et al., 2015a). This ability may be associated with the presence of the *cphA-cphB* operon responsible for the production and degradation of cyanophycin, that is missing in the genome of *Sm* K729a and *Xcc* ATCC 33913. This compound is a branched non-ribosomally synthesized polypeptide that accumulates in cyanobacteria and proteobacteria (Allen et al., 1980; Krehenbrink et al., 2002) and acts as a temporary nitrogen and carbon reserve (Li et al., 2001; Krehenbrink et al., 2002). Therefore, *L. capsici* members could have the genetic basis to promptly adapt to environments lacking in nutrients. *L. capsici* AZ78 is able to resist UV-light irradiation (Puopolo et al., 2014a) and this ability may be associated with the presence of genes involved in the biosynthesis of xanthomonadin, in agreement with the production of a xanthomonadin-like aryl polyene group reported for *L. enzymogenes* OH11 (Wang et al., 2013). *L. capsici* AZ78 has a pronounced resistance to copper ions, which renders this BCA a prime candidate for combination with copper-based fungicides for more efficient control of *Pl. viticola* on grapevine plants (Puopolo and Pertot, 2014). Resistance to copper frequently arises in phytopathogenic xanthomonads (Stall et al., 1986; Behlau et al., 2011), and resistant *Stenotrophomonas* strains have been isolated from copper-polluted soils (Altimira et al., 2012). Some of the genes involved in the resistance to copper are shared among the *Lc* AZ78, *Sm* K729a and *Xcc* ATCC 33913 genomes, whereas genes encoding copper exporting ATPases are specific for *Lc* AZ78 genomes. Based on these differences, *L. capsici* members seem to have the genome makeup necessary to guarantee better survival in an environment with greater concentrations of copper. *L. capsici* AZ78 is also resistant to other heavy metals such as cobalt and zinc and this phenotype is associated with the presence of the *czcCBA* operon and several genes involved in the efflux of cadmium, cobalt and zinc. Most of these genes encode RND proteins known as key multidrug efflux transporters for resistance to antibiotics, dyes, fungicides and solvents in Gram-negative (Kumar and Schweizer, 2005; Bazzini et al., 2011; Yamaguchi et al., 2015). Accordingly, the presence of a high number of RND proteins and other efflux systems (SMR, MFS, and MTE) is also associated with the *Lc* AZ78 resistance to fungicides and insecticides *in vitro*. Regarding resistance to antibiotics, *Lc* AZ78 was sensitive to chloramphenicol and erythromycin although several genes involved in antibiotic resistance are shared with *Sm* K729a. Indeed, this opportunistic human pathogen is resistant to these antibiotics, making it very risky for treating infections in immunocompromised patients (Crossman et al., 2008; Ryan et al., 2009). Thus, it is conceivable that *Sm* K729a genome has genes involved in chloramphenicol and erythromycin resistance with no orthologs in *Lc* AZ78 genome.

In conclusion, the sequence and annotation of the *Lc* AZ78 genome provide a genetic framework for detailed analysis of potential biocontrol mechanisms against phytopathogens. In particular, the comparison of *Lc* AZ78, *Sm* K729a, and *Xcc* ATCC 33913 genomes allows to state that *Lc* AZ78 is missing of the genetic information needed to establish pathogenic interaction with humans and plants, an aspect that is crucial for the registration of new BCAs. This comparative approach highlights the genetic basis determining the *Lc* AZ78 aptitude to compete with phytopathogenic microorganisms through the release of (i) extracellular lytic enzymes; (ii) secondary metabolites with antibacterial and antifungal activity; and (iii) catechol siderophores. Furthermore, the *Lc* AZ78 genome contains a vast number of genes involved in resistance to environmental stress, antibiotics, heavy metals and plant protection products. Analysis of the *Lc* AZ78 genome will help to provide more accurate characterization of bacterial strains belonging to the *Lysobacter* genus and lead to important advances in the further development of *Lc* AZ78 as an active ingredient in new biofungicides.

## Author contributions

GP conceived the work, designed the experiments, carried out annotation of the genome and the experiments, analyzed the data, and wrote and edited the manuscript. ST carried out annotation of the genome and the experiments, analyzed the data, and wrote and edited the manuscript. PS, MM, and KE assembled the genome, wrote and edited the manuscript. MP and IP contributed to the conception of the work, designed the experiments and edited the manuscript. All the authors have read the manuscript and agree with its content.

### Conflict of interest statement

The authors declare that the research was conducted in the absence of any commercial or financial relationships that could be construed as a potential conflict of interest.
