# Supplementary material for: The Lysobacter capsici AZ78 Genome Has a Gene Pool Enabling it to Interact Successfully with Phytopathogenic Microorganisms and Environmental Factors
Source: Front Microbiol. 2016 Feb 5;7:96. doi: 10.3389/fmicb.2016.00096 (PMC4742617; doi:10.3389/fmicb.2016.00096)
Supplement: Supplementary file 1 [file Table1.DOCX]

**Supplementary Table ST1:** **Bacterial and fungal strains used to assess the production of antibacterial and antifungal compounds by *Lysobacter capsici* AZ78.**

| **Phylum** | **Strain** |
| --- | --- |
| Bacteria | *Agrobacterium tumefaciens* |
|  | *Erwinia carotovora* subsp. *carotovora* |
|  | *Ralstonia solanacearum* |
|  | *Xanthomonas campestris* pv. *campestris* DSM 3586 |
|  | *Clavibacter michiganensis* subsp. *michiganensis* LMG 3690 |
|  | *Clavibacter michiganensis* subsp. *sepedonicus* LMG 3690 |
|  | *Rhodococcus fascians* LMG 3605 |
|  | *Streptomyces turgidiscabies* DSM 41838 |
| Fungi | *Alternaria alternata* |
|  | *Ascochyta rabiei* |
|  | *Aspergillus flavus* |
|  | *Aspergillus niger* |
|  | *Aspergillus ochraceus* |
|  | *Botrytis cinerea* |
|  | *Colletotrichum gloeosporioides* |
|  | *Fusarium acuminatum* |
|  | *Fusarium avenaceum* |
|  | *Fusarium oxysporum* f. sp. *asparagi* |
|  | *Fusarium oxysporum* f. sp. *lycopersici* |
|  | *Fusarium oxysporum* f. sp. *radicis-lycopersici* |
|  | *Fusarium sambucinum* |
|  | *Fusarium semitectum* |
|  | *Fusarium solani* |
|  | *Penicillium* sp. |
|  | *Phoma tracheiphila* |
|  | *Pyrenochaeta lycopersici* |
|  | *Rhizoctonia solani* |
|  | *Sclerotinia maior* |
|  | *Sclerotinia minor* |
|  | *Sclerotinia sclerotiorum* |
|  | *Thielaviopsis basicola* |
