# Supplementary material for: The Lysobacter capsici AZ78 Genome Has a Gene Pool Enabling it to Interact Successfully with Phytopathogenic Microorganisms and Environmental Factors
Source: Front Microbiol. 2016 Feb 5;7:96. doi: 10.3389/fmicb.2016.00096 (PMC4742617; doi:10.3389/fmicb.2016.00096)
Supplement: Supplementary file 2 [file Table2.docx]

**Supplementary Table ST2:** **Fungicides and insecticides used to determine the resistance of *Lysobacter capsici* AZ78 to plant protection products.**

| **Active Ingredient (AI)** | **Commercial Product** | **Target**  **Grapevine Disease** | **Dose**  **(AI ml or g /hl)** |
| --- | --- | --- | --- |
| Azoxystrobin | Amistar | Downy mildew | 23.2 ml/hl |
| Copper oxychloride + Iprovalicarb | Melody-Compact |  | 124.6 + 14.7 g/hl |
| Cyazofamid | Mildicut |  | 10.4 ml/hl |
| Cymoxanil | Cymbal 20 |  | 22.8 g/hl |
| Dimetomorph | Quantum |  | 25.3 g/hl |
| Dithianon | Delan 70 WG |  | 84.0 g/hl |
| Folpet | Solofol |  | 121.2 g/hl |
| Folpet + Iprovalicarb | Melody Care |  | 93.75 + 16.5 g/hl |
| Fluopicolide | Profiler |  | 11.0 g/hl |
| Metalaxil-m | Ridomil Gold Sl |  | 9.8 g/hl |
| Metiram | Polyram DF |  | 142.4 g/hl |
| Phosethy-Al | Prodeo 80 WG |  | 200 g/hl |
| Zoxamide | Zoxium 240 SC |  | 16.35 ml/hl |
| Boscalid | Cantus | Grey mould | 60.6 g/hl |
| Cyprodinil + Fludioxonil | Switch |  | 30.0 + 20.4 g/hl |
| Fenexamid | Teldor |  | 75.5 g/hl |
| Fluazinam | Ohayo |  | 59.3 ml/hl |
| Pyrimethanil | Scala |  | 74.8 ml/hl |
| Bupimirate | Nimrod 250 EW | Powdery mildew | 35.7 ml/hl |
| Cyflufenamid | Cidely |  | 2.6 ml/hl |
| Meptyldinocap | Karanthane 3D |  | 21.4 ml/hl |
| Metrafenone | Vivando |  | 10.6 ml/hl |
| Penconazole | Support 10 EC |  | 5.1 ml/hl |
| Quinoxifen | Arius |  | 6.8 ml/hl |
| Spiroxamine | Batam |  | 20.2 ml/hl |
| Tebuconazole | Fezan |  | 9.87 ml/hl |
| Sulphur | Thiamon 80 Plus |  | 565.6 g/hl |
| Chlorantraniliprole | Coragen | European grapevine moth | 3.3 ml/hl |
| Chlorpyriphos | Dursban |  | 53.0 g/hl |
| Emamectin benzoate | Affirm |  | 1.4 g/hl |
| Spinosad | Laser |  | 8.4 ml/hl |
| Thiamethoxam | Actara 25 WG |  | 5.1 g/hl |
